# Comparative study of fecal calprotectin levels in ulcerative colitis versus irritable bowel syndrome

**DOI:** 10.6026/973206300211850

**Published:** 2025-07-31

**Authors:** Geethika Sai Satyavasukiaparna Garaga, Kalvakota Maninithya, Nadampalli Deepica Rani, Aksah Dayan

**Affiliations:** 1 Department of Gastroenterology and Hepatology, Yashoda Hospitals, Hyderabad, Telangana, India; 2 Department of Medical Gastroenterology, Yashoda hospital, Hyderabad, Telangana, India; 3 Department of General Medicine, Yashoda hospitals, Hyderabad, Telangana, India

**Keywords:** Fecal calprotectin, ulcerative colitis, irritable bowel syndrome, inflammatory bowel disease, non-invasive biomarker, ELISA

## Abstract

Fecal calprotectin has been evaluated on its ability to differentiate between ulcerative colitis (UC) and irritable bowel syndrome (IBS). Hence, sixty patients were stratified according to clinical and histological characteristic as UC (Group A) and IBS (Group B). The levels of calprotectin in UC patients (623.5 0 g) were significantly higher than in IBS patients (36.4 0 g), (p < 0.001). The high presence was observed in 93.3 percent of UC subjects and only 6.7 percent of IBS subjects. Thus, fecal calprotectin works well in distinguishing UC and IBS, and facilitates the non-invasive diagnosis of UC.

## Background:

Many people suffer from ulcerative colitis (UC) and irritable bowel syndrome (IBS) which have similar symptoms such as diarrhea, abdominal pain and urgent bowel movements, making it difficult to diagnose these conditions accurately. In IBS, there is no physical or chemical reason found, but in UC, chronic inflammation is found only inside the colon, causing damage to the intestines if not managed [1, 2]. It is very important to tell apart diabetes mellitus from diabetes insipidus, because their treatments are completely different. Existing methods for testing UC are colonoscopy and viewing histological samples which are proven to be accurate but are invasive, not always convenient for general screening and often costly [3]. Therefore, people are now paying more attention to non-invasive biomarkers that can help doctors tell inflammatory GI diseases apart from those that are not inflammatory. Fecal calprotectin which is mostly collected from neutrophils and binds calcium and zinc has been found to accurately measure intestinal inflammation. You can measure it in feces with ELISA-based tests and it remains stable for a week at room temperature [4]. If you have UC or Crohn's disease, high fecal calprotectin levels suggest there is inflammation, but they are normal in disorders like IBS, showing no inflammation [5, 6]. Several research papers have found that using fecal calprotectin as a test gives high sensitivity and specificity in differentiating inflammatory bowel disease (IBD) from irritable bowel syndrome (IBS). It has also been suggested as a means to monitor a disease's progress, anticipate a future relapse and avoid extra endoscopic procedures [9]. There are not many direct studies of fecal calprotectin in people with UC and IBS under the same clinical situation. Moreover, the biologic process leading to high fecal calprotectin gives good reason for using it to diagnose inflammation. Active ulcerative colitis causes neutrophils to enter the colon lining which results in calprotectin being released into the stool and making its concentration very high [10]. That is, IBS does not cause inflammation or a rise in neutrophils, so calprotectin levels usually stay normal. This difference can be used to tell inflammatory and functional problems in the gut by doing a simple stool test. In recent times, calprotectin in stool samples is now commonly used to help decide when to refer patients for further investigation. Clinicians can use it to decide who needs a colonoscopy if there is suspicion of inflammatory bowel disease, but no official confirmation has been made [11]. Fecal calprotectin testing is recommended by the National Institute for Health and Care Excellence (NICE) to help prevent unneeded endoscopic tests in patients who have lower gastrointestinal issues [12]. So, carrying out fecal calprotectin testing could make it easier for patients to be diagnosed and treated on time, lower total healthcare expenses and help patients in UC achieve better outcomes. Even though fecal calprotectin is very useful, there are some restrictions to consider. Using N SAIDs, having a gastrointestinal infection or having colorectal tumors may cause false positives due to mucosal inflammation [13]. It is also important to mention that the values considered high are variable, nonetheless most studies use >50 µg/g as significant. Fecal calprotectin is considered together with clinical findings and other medical tests. Therefore, it is of interest to report the comparative study of fecal calprotectin levels in ulcerative colitis versus irritable bowel syndrome.

## Materials and Methods:

There were 60 patients aged 18 to 60 years, who were sorted into two groups by following the established diagnostic rules. Group A had 30 patients who were diagnosed with ulcerative colitis (UC) after experiencing typical symptoms, along with insights from colonoscopy and biopsy reports. Group B included 30 people who were diagnosed with irritable bowel syndrome (IBS) by Rome IV, but had normal colonoscopy and biopsy results. Participants wrote and signed to inform us that they were informed of the study details. Patients with other problems in the gastrointestinal tract (like infections or malignancies), those who recently took NSAIDs or antibiotics and those who have had gastrointestinal surgery were not included to make the results more reliable. Every participant had a fresh stool sample placed in a sterile container. Within 24 hours, the samples were processed and the fecal calprotectin concentrations were tested by using an ELISA kit made by a company, as the manufacturer directed. Any fecal calprotectin result above 50 µg/g was thought of as elevated. Standard procedures for collecting stool samples were explained to patients to make sure there was no difference in the samples. Samples were kept at about 2-8°C before being taken to the laboratory for homogenization and splitting into aliquots for ELISA analysis. The results were more accurate and reliable because each test was done twice. Those involved in the lab analysis were unaware of the grouping so their decisions would not be affected by knowledge of the patient's illness. Each person's case was documented according to length of symptoms, medicine taken and other related conditions. Fecal calprotectin levels were related to age and sex using Pearson correlation coefficient and chi-square test respectively. It made it possible to check if the primary outcome was affected by the participants' demographics. Applying rigorous procedure for sample management, carrying out tests and analyzing data made the comparative findings of the study between the groups of ulcerative colitis and irritable bowel syndrome sturdy. Demographic data such as age and gender, as well as clinical history, were documented. Statistical analysis was performed using SPSS software version 25.0. Mean values and standard deviations were calculated for continuous variables and proportions for categorical variables. Comparison between groups was done using the independent samples t-test. A p-value of less than 0.05 was considered statistically significant.

## Results:

A total of 60 patients were enrolled in the study, with 30 individuals in each group: ulcerative colitis (UC) and irritable bowel syndrome (IBS). The mean age of participants in the UC group was 39.2 ± 11.6 years, while in the IBS group it was 36.7 ± 9.4 years, with no statistically significant difference (p = 0.32). Gender distribution was comparable between the two groups (Table 1 - see PDF). Fecal calprotectin levels were significantly higher in the UC group compared to the IBS group. The mean fecal calprotectin concentration in the UC group was 612.8 ± 190.5 µg/g, whereas the IBS group had a mean level of 38.2 ± 14.6 µg/g (p < 0.001), indicating a strong distinction between inflammatory and functional bowel disease (Table 2 - see PDF). Elevated fecal calprotectin levels (>50 µg/g) were observed in 28 out of 30 patients (93.3%) in the UC group, while only 2 patients (6.7%) in the IBS group had mildly elevated levels. There was no statistically significant association between fecal calprotectin levels and age or gender in either group. As shown in Table 2 (see PDF), the marked difference in fecal calprotectin levels between UC and IBS supports its utility as a non-invasive tool in clinical differentiation of these conditions.

## Discussion:

Our study confirms that patients with ulcerative colitis (UC) have considerably elevated fecal calprotectin levels when compared to those with irritable bowel syndrome (IBS), strengthening its usefulness for diagnosing different bowel disorders. The average fecal calprotectin reading for UC cases was above 600 µg/g, but for patients with IBS, most of the readings were under 50 µg/g. earlier research has also shown that high fecal calprotectin indicates intestinal inflammation in people with IBD [1, 2]. In variable amounts, neutrophils release fecal calprotectin when the intestinal lining is inflamed and it serves to indicate how many neutrophils travel into the gut [3, 4]. That biological process is believed to explain why neutrophils are found in higher numbers in UC patients, where this is a distinguish feature seen under the microscope [5]. Unlike these other disorders, IBS is considered a functional gastrointestinal disease with no inflammation in the digestive tract, so it typically does not show high levels of calprotectin in the stools [6, 7-8]. The fact that this test is non-invasive supports the trend in gastrointestinal testing to value patient comfort, cost savings and still achieve high diagnostic accuracy [9]. If a patient has UC, their doctor may use fecal calprotectin testing to both understand what is happening in their body and predict upcoming relapses [10]. A decrease in calprotectin levels is linked to improved (less) endoscopic and histological inflammation and when values are back to normal, it often correlates with healed mucosa [11, 12]. Because of this, doctors can determine how well a patient is responding and modify their treatment plan without the need for invasive tests. Even though it has many advantages, fecal calprotectin testing does also have some limitations. Some people might have high levels in infections of the colon, colon cancer or side effects from taking nonsteroidal anti-inflammatory drugs [13]. That is why, when a case is unclear, doctors must rely on their experience and further testing. Also, because every laboratory has different cut-off levels and testing routines, the results are not always the same, so it is important to use standard guidelines [14, 15]. This study confirms that fecal calprotectin can help doctors distinguish UC from IBS in patients who have similar gastrointestinal complaints. That there are no major correlations found with age or gender further supports that it can be applied to many groups of patients. Still, numerous studies including patients from many centers are needed to test these findings and look into calprotectin's role in early detection and on-going disease monitoring.

## Conclusion:

Checking fecal calprotectin often helps doctors when deciding between UC and IBS. When used in everyday clinical works, it helps improve the way diagnosis is made, therapies are chosen and the need for invasive exams is reduced.
